# The Na_v_ channel bench series: Plasmid preparation^☆^

**DOI:** 10.1016/j.mex.2014.01.002

**Published:** 2014-02-01

**Authors:** Daniel H. Feldman, Christoph Lossin

**Affiliations:** Department of Neurology, University of California - Davis, United States

**Keywords:** Sodium channel, Na_v_ channel, Mutation, Plasmid, DNA, Na_v_1.1, *SCN1A*, Na_v_1.6, *SCN8A*, Stbl2, Channelopathy

## Abstract

Research involving recombinant voltage-gated sodium (Na_v_) channels has unique challenges. Multiple factors contribute, but undoubtedly at the top of the list is these channels’ DNA instability. Once introduced into bacterial hosts, Na_v_ channel plasmid DNA will almost invariably emerge mutagenized and unusable, unless special conditions are adopted. This is particularly true for Na_v_1.1 (gene name *SCN1A*), Na_v_1.2 (*SCN2A*), and Na_v_1.6 (*SCN8A*), but less so for Na_v_1.4 (*SCN4A*) and Na_v_1.5 (*SCN5A*) while other Na_v_ channel isoforms such as Na_v_1.7 (*SCN9A*) lie in between. The following recommendations for Na_v_ plasmid DNA amplification and preparation address this problem. Three points are essential:

• Bacterial propagation using Stbl2 cells at or below 30 °C.
• Bias toward slow-growing, small bacterial colonies.
• Comprehensive sequencing of the entire Na_v_ channel coding region.

## Graphical abstract

**Figure:**
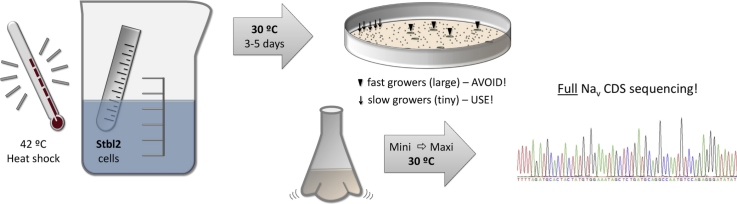

## Method details

### Step 1: transformation

#### Materials

• high quality starter Na_v_ plasmid DNA
• Max Efficiency^®^ Stbl2 competent cells (Life Technologies)
• LB plates (with antibiotic at half-standard concentration)
• SOC or comparable outgrowth medium [8]
• dedicated 30 °C incubator and shaker
  *Note*: This list includes only non-standard items. Generic components such as water baths and baffled flasks are assumed to be available.

Analyses of Na_v_ channel function begin with the construction or purchase of an expression vector (e.g., pCMV-Scrip, pCI-neo) containing a Na_v_ channel's coding sequence. Alternative splicing is common among Na_v_ channels, creating different proteins from the same gene with distinct spatial and temporal expression patterns [9–12]. Careful planning is therefore necessary to determine exactly which isoform and coding sequence are needed to address the questions of interest. With approximately 6 kb in length, Na_v_ channel coding sequences are long, making final insert-vector plasmid construct sizes in excess of 11 kb common. Regardless of its origin, be it a gift from a colleague or purchase from a commercial supplier, comprehensive sequence information ought to be part of the plasmid construct transaction. This will allow the end user to be fully informed when it comes to restriction analysis of the plasmid, which should always be the first step upon the DNA's arrival. Simple electrophoresis of small aliquot of plasmid DNA digested with a reliable enzyme that produces a known banding pattern will show whether the plasmid is correct or not. This may seem trivial, but cases where providers less experienced with Na_v_ channel handling send corrupted DNA are not uncommon. If above mentioned restriction digest does not deliver the expected results (be selective here; minimal changes can be telltale signs of problems), digest with an alternative enzyme to confirm.

Once the restriction enzyme digestions delivered the appropriate restriction fingerprint, bacterial transformation can begin. We have had good success with chemical transformation as described below, but electroporation of suitable bacterial strains may be a viable alternative. Contrary to standard transformations, competent cell choice is critical when comes to Na_v_ channels. Among the cell lines known to be susceptible to produce problems are JM101, DH5α, One Shot Top10^®^, to name but a few. Transforming these cells according to the manufacturer's instructions has a high chance of producing a negative outcome, although in select cases success appears to be possible (JM109, unpublished data). In our hands, by far the most reliable and successful is the Stbl2 bacterial strain, whose genotype favors cloning of problematic plasmid DNA. Although somewhat costly, it is possible to refreeze them at −80 °C in 25-μl ready-to-use aliquots using immediate immersion in ethanol/dry ice without losing too much efficiency, should a more economical solution be necessary.

Once the transformation is completed, the bacteria are propagated on LB plates with an appropriate antibiotic, at approximately half-standard concentration (e.g., 50 vs. 100 μg/ml ampicillin), at 27–30 °C. Because this temperature is lower than the 37 °C incubation used in standard procedures, using a lower concentration of antibiotic will allow faster bacterial growth without compromising the selection of resistant clones. It is very important at this point to emphasize the use of a dedicated and appropriately labeled 30 °C incubator. Any temperature elevation of Stbl2 cells above 30 °C will inevitably corrupt Na_v_ channel DNA through host-mediated mutation (personal observation). At this temperature, the growth of bacteria harboring Na_v_ channel DNA is very slow, and incubations of 3–5 days are often necessary. At no time during this period can the temperature exceed 30 °C. Appropriate signage ought to be attached to the incubators to ward off secondary users who may briefly change the incubator temperature to the standard 37 °C unbeknownst to the Na_v_ channel researcher.

#### Procedure

• ice-chill 2 μl (20 ng) of plasmid DNA in the bottom of a Falcon tube
• thaw a 25-μl aliquot of Stbl2 cells on ice for 5 min, then pipette over the chilled DNA and mix with gentle pipette tip agitation
• incubate on ice for 25 min, then heat shock in a 42 °C water bath for 40 s and immediately return to ice for 2 min
• add 400 μl of room-temperature SOC and shake in a pre-warmed incubator at 30 °C for 90 min
• plate 50 and 250 μl of the cells on two separate, pre-warmed LB plates with antibiotic at half-standard concentration
• following a 10-min right-side up absorption at room temperature, place the plates upside-down in the dedicated 30 °C incubator
• allow to incubate for several days until two distinct populations of colonies have emerged: large fast-growers and tiny slow-growers

### Step 2: bacterial growth

#### Materials

• dedicated 30 °C incubator/shaker
• 1 l LB broth (selective antibiotic at half-standard concentration)
  *Note*: As in Step 1, this list includes only non-standard items. Also required are commercial plasmid prep kits (mini as well as maxi), gel electrophoresis instrumentation, and associated consumables.

As mentioned earlier, the success of the transformation will not be known for several days. Although colonies might be visible the morning after the first overnight incubation, these fast growing colonies very likely carry corrupted plasmid DNA and should be avoided. In the cases that we have examined, the extent of the associated genetic alterations in fast growing colonies varied greatly, ranging from point mutations to large-scale rearrangements. The best approach is to wait for 2–3 days before the plates are scanned for colony growth, in which time, a second, slow-growing population of exceedingly small colonies will become discernible. To make colony spiking easier, incubation at 30 °C or room temperature may continue for up to 5 days. Care should be taken when ampicillin is used as the selective antibiotic. Rapidly growing resistant colonies will secrete beta-lactamase that can allow growth of non-resistant cells, which will appear as small, slowly-growing satellites surrounding fast-growing colonies. Bias toward slowly-growing, well-separated colonies helps avoid this issue. An alternative approach is to use an antibiotic such as for tetracycline for which resistance is only effective in the cells that express the resistant gene.

We recommend spiking 10–20 colonies to generate standard LB mini-cultures; the concentration of the selective antibiotic should be identical to that of the previously used plate. Shaking proceeds at standard speeds in standard round-bottom tubes. Maintenance of the 30 °C rule is crucial, which translates into 2–3 days of growth to reach the desired density, although shorter incubation times have been observed. We have experimented with richer media such as terrific broth, but the results were mixed. Terrific broth does accelerate bacterial growth, but it is also more prone to overgrow, which creates downstream problems in plasmid isolation if the growth is not stopped in time.

Once the cultures have reached the appropriate turbidity, they are processed with any standard mini plasmid prep kit since no one commercial kit is advantageous over another. However, it is important to use the maximal amount of cell material allowable per manufacturer's instructions; we commonly process 1.5 ml of culture. The eluted DNA is subjected to the same restriction analysis employed upon arrival of the original DNA. For this digest, simple mixing of the eluate with restriction buffer and enzyme (without addition of water) should yield the best results, as the DNA concentration is likely very low. Alternatively, a smaller elution volume may be used to achieve more concentrated DNA samples.

Provided the mini culture analysis generated the expected restriction pattern, a maxi culture can be started. We commonly increase the manufacturer's recommended culture volume by a factor of 2 or 3 for the culture setup. In other words, if a 200-ml culture is suggested, we usually grow three baffled flasks of 200 ml LB with the aforementioned half-standard antibiotic concentration. Inoculation is done at 1:1000 by adding 200 μl of the original mini-culture (kept at 4 °C) per flask. Best practice is to process the mini-preps and inoculate the maxi-cultures on the same day.

Contrary to all previous incubations, the maxi cultures, while also grown at 30 °C, usually grow quicker and reach sufficient turbidity after only an overnight shake. One can now process the cultures according to the manufacturer's recommendations. We do recommend individual processing of each maxi culture to avoid clogging of the filtration columns after alkaline lysis. However, the DNA from all three setups can be combined in the final step by serial passage of the eluate from one column to the next. Alternatively, one could simply combine the three eluates and reduce their volume in a speedvac, granted that water was used in the elution step.

#### Procedure

• following several days of growth, inspect the LB plates and identify suitable slow-growing, small colonies
• using autoclaved toothpicks, inoculate several 3-ml LB mini cultures (half-standard antibiotic concentration)
• shake at 30 °C for 2–3 days until the cultures turn turbid
• process 1.5 ml of mini cultures according to the instructions of the available mini prep kit using water in the final elution step; store the remainder of the mini culture at 4 °C
• using only restriction buffer, enzyme, and eluate, set up 20-μl digests and separate with standard gel electrophoresis
• identify a setup with the expected restriction fingerprint and 1:1000 inoculate two to three 200-ml maxi cultures (LB, half-standard antibiotic concentration) with 200 μl of mini culture
• shake at 30 °C over night
• process each 200-ml culture separately according to the maxi prep kit manufacturer instructions; only the final TE buffer elution step is customized: combine the DNA yield by sending the eluate from the first silica column through the second (and the third, if three maxi cultures were processed); intermittent volume adjustment of the eluate with additional TE buffer may be necessary

### Step 3: DNA sequencing

Once the maxi prep DNA has been obtained, standard spectrophotometric analysis will help determine the DNA concentration, and restriction fingerprinting as described earlier will confirm the isolated DNA's identity. Despite all optimization efforts, the DNA yield may be low, which may interfere with DNA sequencing. Service facilities frequently request plasmid concentrations in excess of 1 μg/μl for double-stranded DNA larger than 10 kb. Therefore, the initial elution volume can be kept small to achieve higher concentration of working DNA, which can then be diluted to the desired level if needed. Moreover, it is our experience that close communications with the sequencing facility staff can also help address these issues. With additional care and informed personnel, we have not had any problems sequencing our Na_v_ channel plasmids, even at concentrations as low as 100 ng/μl.

Full-length sequencing encompassing the entire Na_v_ coding region is essential. With today's DNA sequencing technology, primer walking with 600–800 bp intervals ought to generate reliable results. We additionally process our chromatograph data using Nucleics’ online PeakTrace application, which aids in clearing base-calling ambiguities. All sequencing data is routinely aligned to our reference sequence (preferably an NM RefSeq or LRG record – hyperlinks provided in Additional information section) where an experienced reviewer visually confirms all base calls. Questionable areas ought to be re-sequenced and, if necessary, primed in both directions. We provide a list of sequencing primers in the online material of this article. Once the Na_v_ channel DNA has been certified in this fashion, it can be used for transfection and/or mutagenesis for downstream analysis of the channel function.

## Additional information

### Background

Voltage-gated sodium (Na_v_) channels control excitation in brain, heart, and muscle. Deviations from their tightly defined functional characteristics, for example through genetic alterations, lead to heritable pathology that reflects the spatial and temporal expression pattern of the affected Na_v_ channel gene ([1–7], reviewed in [13]). Pharmacological control of the clinical symptoms in these so-called Na_v_ channelopathies (e.g., epilepsy, cardiac arrhythmia, muscle cramps, pain) is often hampered by treatment-emergent adverse effects due to structural and functional similarities among the Na_v_ channels that make isoform-specific targeting difficult. Improved intervention may be possible with patient-tailored therapy, where the drug action counters the defect of the faulty Na_v_ isoform without affecting other Na_v_ channel function. Such drug development efforts are challenged by the unique requirements that come with recombinant DNA work involving Na_v_ channel coding sequences, because Na_v_ channels are notoriously difficult subjects when it comes to bacterial plasmid amplification, mutagenesis, and stable cell line development. The basis for this behavior is unknown, and no study researching the same has been published. One may speculate that the combination of exceptionally large coding sequences with internal homologous repeats is involved. There is evidence that Na_v_ channel DNA in itself is toxic for the bacterial host, since random mutation events are not limited to full-length Na_v_ channel coding sequences, but they also occur during bacterial propagation involving Na_v_ channel DNA fragments (personal observation).

### Useful links

• PeakTrace software – www.nucleics.com/peaktrace
• RefSeq: The NCBI Reference Database – www.ncbi.nlm.nih.gov/refseq
• LRG: Locus Reference Genomic – www.lrg-sequence.org

## Appendix A Supplementary data

Further information on Na_v_1.1 sequencing primers is provided as supplementary material.

Supplementary Data - MethodsX 1_6, 2014.
