# Supplementary material for: The Nav channel bench series: Plasmid preparation
Source: MethodsX. 2014 Feb 1;1:6–11. doi: 10.1016/j.mex.2014.01.002 (PMC4472833; doi:10.1016/j.mex.2014.01.002)
Supplement: Supplementary file 1 — Supplementary Data - MethodsX 1_6, 2014. [file mmc1.pdf]

Table 1: Na_v1.1 sequencing primers.

Name	5' - Sequence - 3'	Length (Bases)
**hNav1.1-620F**	TGGACCTGGGCAATGTCTCG	20
**hNav1.1-1017**	CTCTGATGCAGGCCAATGTC	20
**hNav1.1-1467F**	GAGTTCCAAGAGTGCTAAGG	20
**hNav1.1-1734F**	AGGGCGAGCAAAGGATGTGG	20
**hNav1.1-1833R**	GGGCACAAACAAGGAATCTC	20
**hNav1.1-2316F**	GGCCATCACCATCTGTATTG	20
**hNav1.1-3046F**	GTGGATAGGATGCACAAAGG	20
**hNav1.1-3607F**	CAATGGTGGAACCTGAGAAG	20
**hNav1.1-4337F**	ATGTGGAACTCCAGCCTAAG	20
**hNav1.1-4896F**	TACCCTGTTCCGAGTGATCC	20
**hNav1.1-5442F**	GTTTGATCCCGATGCAACTC	20
**hNav1.1-6356R (3' UTR)**	TGGATCTCCATTCGCCATTC	20

Primer identifiers are made up of the protein name and a numeral that stands for the primer's first 5' base with respect to the open reading frame position. The letters "F" and "R" indicate forward and reverse priming, respectively. The tables in this article do not claim to be comprehensive and serve only as recommendations. Regions that have proven to be problematic may be covered by several primers. Upstream sequencing with a vector primer may be necessary where reverse priming of the reading frame 5' end is not provided. Owing to the research focus in our laboratory, we address only select human Na_v channels (Na_v1.1, Na_v1.2, Na_v1.4, Na_v1.5, Na_v1.6, and Na_v1.7). Note that, due to sequence similarities between the different Na_v channels, some of the primers can be used on several different isoforms. For Na_v1.1, this includes hNav1.2-656F, hNav1.7-2576F, hNav1.7-4802R, and hNav1.2-4972F.

Table 2: $Na_v1.2$ sequencing primers

Name	5' - Sequence - 3'	Length (Bases)
**hNav1.2-319R**	GGGTGGCACTGAATCGAGAG	20
**hNav1.2-656F**	TCAGAGTTCTCCGAGCATTG	20
**hNav1.2-783R**	CACGCTTAGACAGAACACAG	20
**hNav1.2-1278F**	GGCCTATGAGGAACAGAATC	20
**hNav1.2-1840F**	CCGCACAGACATGGAGAACG	20
**hNav1.2-2325F**	CATGGCTATGGAGCACTATC	20
**hNav1.2-2863F**	GGCCAAACCATGTGCCTTAC	20
**hNav1.2-3420F**	ATCTGAAGGCAGCACGGTTG	20
**hNav1.2-3952F**	TCCCGGTTTGAAGGAATGAG	20
**hNav1.2-4190R**	CTGGCAGTTTGATTGCTCTC	20
**hNav1.2-4535F**	CCATACCTCGACCTGCTAAC	20
**hNav1.2-4972F**	CCTGCGTTGTTTAACATCGG	20
**hNav1.2-5421F**	CGATGCGACCCAGTTTATAG	20

Primers not originally intended but functional for $Na_v1.2$ include hNav1.7-4802R and hNav1.1-4896F (Table 6 and 1, respectively).

Table 3: Na$_v$1.4 sequencing primers

Name	5' - Sequence - 3'	Length (Bases)
**hNav1.4-370F**	GCCATCAAGGTGCTCATCC	19
**hNav1.4-500R**	ATCCCTGTGAAGGTGTACTC	20
**hNav1.4-855F**	GCCGTTCAACGACACCAACAC	21
**hNav1.4-1386F**	GGAGAAAGAGGAGGAGTTTCAG	22
**hNav1.4-1832F**	CTGTGGGCAACCTGGTCTTC	20
**hNav1.4-2360F**	TCATCGGCAATCTTGTGGTC	20
**hNav1.4-2894F**	GCAGCACAGCTGACTACAAG	20
**hNav1.4-3372F**	CATCAAATCCCTGCGGACAC	20
**hNav1.4-3856F**	ACCCTCAACCTCTTCATTGG	20
**hNav1.4-4332F**	CACGCTGTTCCGTGTGATCC	20
**hNav1.4-4894F**	ACCCAGTTCATCGCCTACAG	20
**hNav1.4-5330F**	GCAAGATGTATGGCCACGAG	20

Details as explained in Table Legend 1.

Table 4: $Na_v1.5$ sequencing primers

Name	5' - Sequence - 3'	Length (Bases)
**hNav1.5-416F**	GCACCATCCTCACCAACTG	20
**hNav1.5-903F**	GGAATCCCTGGACCTTTACC	20
**hNav1.5-1402F**	CCAGTAAACAGCCATGAGAG	20
**hNav1.5-1776F**	CAGCACTGTGGACTGCAATG	20
**hNav1.5-2443F**	GTCTTCAAGCTGGCCAAATC	20
**hNav1.5-2923F**	CGGACCACCTGGGATTTCTG	20
**hNav1.5-3415F**	GGCAGCACAGCAGACATGAC	20
**hNav1.5-3943F**	TCACGATTTGAGGGCATGAG	20
**hNav1.5-4444F**	GGCCAGGACATCTTCATGAC	20
**hNav1.5-4893F**	CCGCATCCTCAGACTGATCC	20
**hNav1.5-5433F**	TTCGGTCCTGTCTGACTTTG	20

Details as explained in Table Legend 1.

Table 5: $Na_v1.6$ sequencing primers

Name	5' - Sequence - 3'	Length (Bases)
**hNav1.6-317F**	TTAGTGCCACGCCTGCCTTG	20
**hNav1.6-852F**	GCCCATAAACTTCAACGAGAGC	20
**hNav1.6-1374F**	CTCAGAAGATGCCATAGAGG	20
**hNav1.6-1918F**	AACAGCACGGTGGACTGCAACG	22
**hNav1.6-2411F**	TCATAGCCATGGATCCCTAC	20
**hNav1.6-2900F**	TGGTGCTGAACCTGTTTCTG	20
**hNav1.6-3393F**	CTCTGAAGGAAGCACCATTG	20
**hNav1.6-3868F**	GGTGCCATAAAGTCCCTTAG	20
**hNav1.6-4363F**	ACCCTGAACCTGTTCATTGG	20
**hNav1.6-4908F**	GATTCGTACCCTGCTCTTTG	20
**hNav1.6-5412F**	GTACTGTAAGCTGGCAGAC	19

Details as explained in Table Legend 1.

Table 6: $Na_v1.7$ sequencing primers

Name	5' - Sequence - 3'	Length (Bases)
**hNav1.7-680R**	ACAGAAATAGTTTTCAAAGC	20
**hNav1.7-707F**	GGGCTTTGATCCAGTCAGTG	20
**hNav1.7-1029R**	GCTCGTGTAGCCATAATCAG	20
**hNav1.7-1359F**	CAGAATTATGGGCCTCTCAG	20
**hNav1.7-2095F**	TGTCCACCTTGGTGGTACAG	20
**hNav1.7-2276R**	TCAGTCATTGGGTGGTGTTC	20
**hNav1.7-2576F**	TGTTGGCCATCATCGTCTTC	20
**hNav1.7-4038F**	ACGGTTTCCTGCAAGTCAAG	20
**hNav1.7-4222R**	CAGAATCCACTGCTGCATAC	20
**hNav1.7-4228F**	GTAGACAAGCAGCCCAAA	18
**hNav1.7-4802R**	ATCACTCGGAACAGGGTAGG	20

Details as explained in Table Legend 1. Primers not originally intended but functional for $Na_v1.7$ include hNav1.1-4896F (Table 1) as well as hNav1.2-4972F and hNav1.2-5421F (Table 2).
